# Pictorial essay: Mammography of the male breast

**DOI:** 10.4103/0971-3026.57207

**Published:** 2009-11

**Authors:** Manju Bala Popli, V Popli, P Bahl, Y Solanki

**Affiliations:** Department of Radiological Imaging and NMR Research Centre, Institute of Nuclear Medicine and Allied Sciences (INMAS), Lucknow Road, New Delhi - 110 054, India; 1 Aruna Asaf Ali Hospital, Rajpur Road, New Delhi - 110 054, India

**Keywords:** Breast, male, mammography

## Abstract

Mammography is an imaging modality that is widely perceived to be of use only in women for the detection and diagnosis of breast pathologies. Here, we present a pictorial essay on the mammographic spectrum of male breast pathologies.

## Introduction

Mammography is a sensitive and cost-effective imaging modality in women. It is carried out for screening and for diagnostic purposes. The low incidence of breast cancer in males does not warrant screening mammography and, therefore, mammography in males is a diagnostic study.[1–3] Patients with complaints of breast enlargement, tenderness or lump are referred for mammography.

Gynecomastia is the most common cause of breast enlargement. Patients with breast cancer present with a lump rather than breast enlargement. It is critical to distinguish between these two pathologies and mammography has been shown to be a useful imaging modality for the same.[4–6]

## Discussion

**Figure 1 (A,B):**
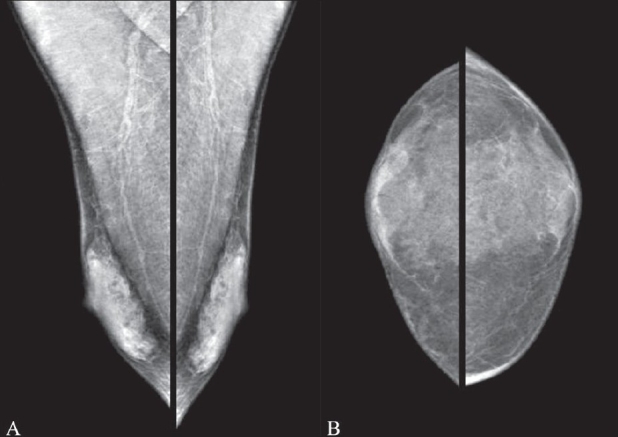
Normal male breast. Mediolateral oblique (A) and craniocaudal (B) views of both breasts show the normal appearance

The normal male breast consists of subcutaneous fat and remnant subareolar ductal tissue [Figure 1]. Lobular development is not seen; hence, lobular processes like lobular carcinoma, adenosis and fibrocystic changes are not seen in males. Here, we discuss the mammographic features of breast pathologies seen in males.

### Gynecomastia

**Figure 2 (A,B):**
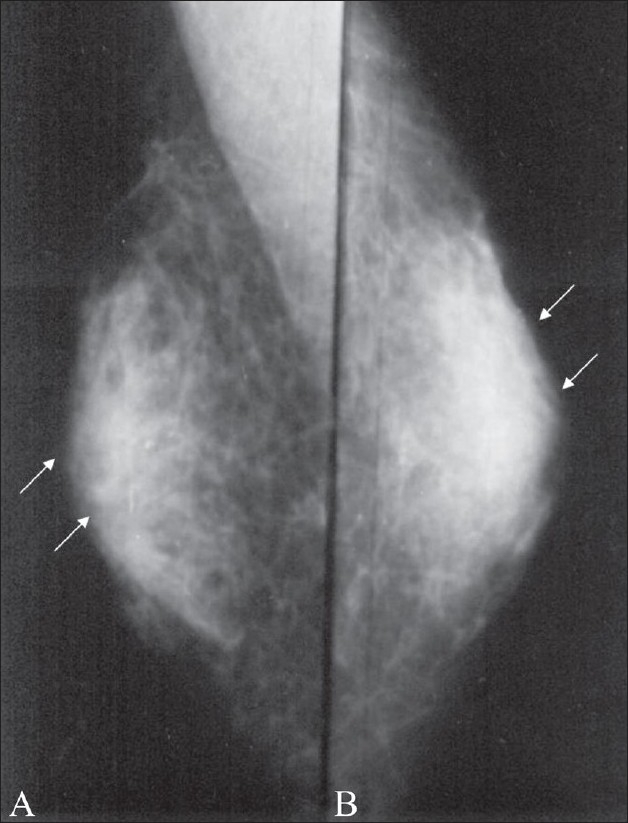
Dendritic gynecomastia. Mediolateral oblique (A) and craniocaudal (B) views of the right breast show dendritic gynecomastia (arrows)

**Figure 3 (A,B):**
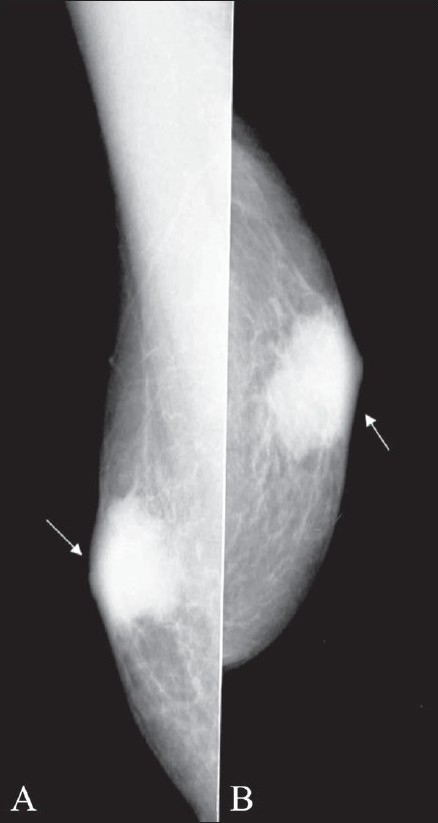
Nodular gynecomastia. Mediolateral (A) and craniocaudal (B) views of the left breast show nodular gynecomastia (arrows)

Gynecomastia is the most common cause of male breast enlargement and is usually bilateral. Patients may however present with unilateral breast enlargement or tenderness. Gynecomastia is characterized by hyperplasia of the stromal and ductal elements in the breast. Clinically, the breast is enlarged, soft and tender and a mass may be palpable in the retro-areolar region. On mammography, gynecomastia can be nodular, dendritic or diffuse.[2–4] Dendritic gynecomastia appears as ‘flame-shaped’ fibroglandular tissue in the retro-areolar area, which radiates from the nipple into the deeper adipose tissue [Figure 2]. It is thought to be a result of long-standing gynecomastia. It is histologically characterized by ductal proliferation, with surrounding fibrotic stroma. In nodular gynecomastia, a nodular, mass-like lesion is seen in the retro-areolar region [Figure 3]. In diffuse gynecomastia, the mammographic appearance is that of a dense breast. There is enlargement of the breast with diffuse density and both dendritic and nodular features may be seen. Absence of a well-defined identifiable mass and the secondary signs differentiate gynecomastia from malignancy.

### Pseudogynecomastia

**Figure 4:**
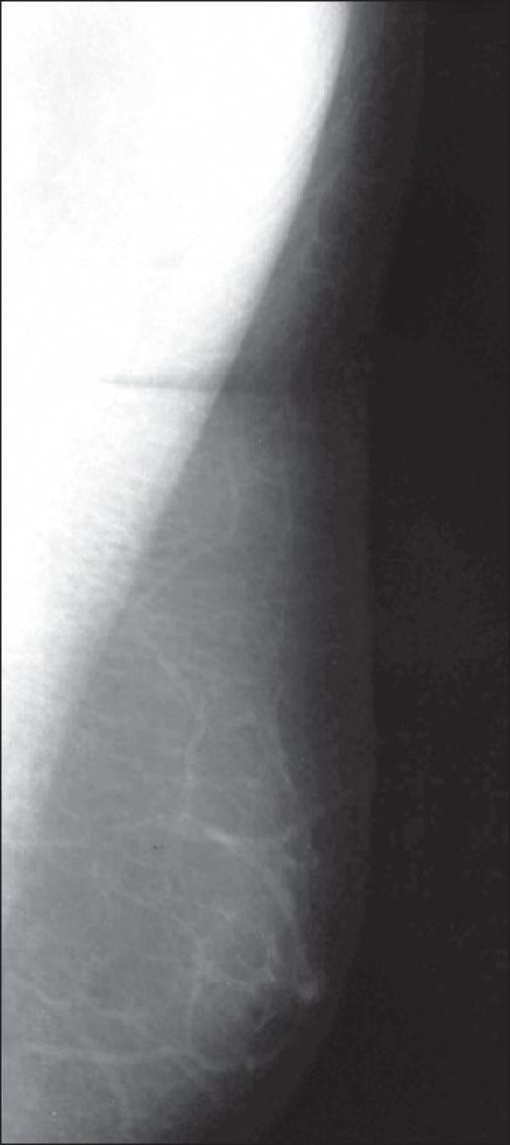
Pseudogynecomastia. Mediolateral view of the left breast shows only fat. There is absence of retrao-areolar ductal opacities, suggestive of pseudogynecomastia

In true gynecomastia there is involvement of the breast tissue. Male breast enlargement, as a result of accumulation of excessive fat tissue with a lack of actual breast tissue, is pseudogynecomastia [Figure 4]. This condition is common in older men and overweight young men.

### Retro-areolar abscess

**Figure 5 (A,B):**
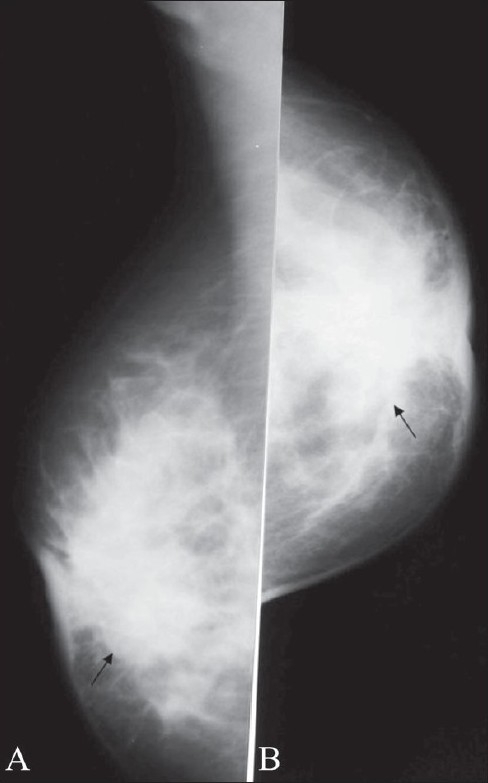
Retro-areolar abscess. Mediolateral (A) and craniocaudal (B) views of the breast show a retro-areolar abscess (arrows)

On mammography, a retro-areolar abscess appears as a mass with indistinct margins [Figure 5]. There is thickening of the skin. The lesion can be mistaken for carcinoma breast.

### Epidermal inclusion cyst

**Figure 6:**
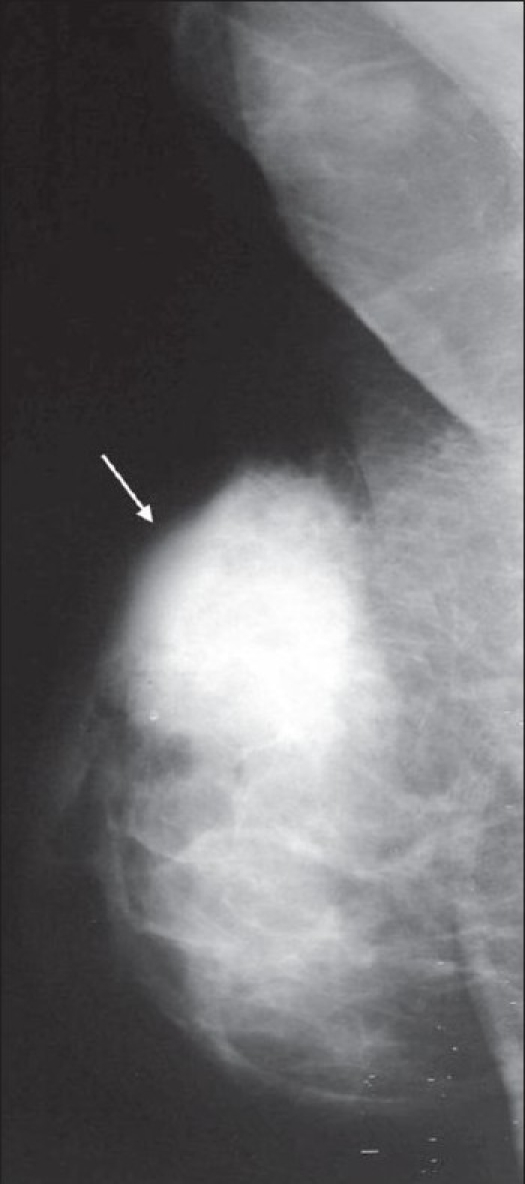
Epidermal inclusion cyst. A well-defined space-occupying lesion is seen in this mediolateral view (arrows). This turned out to be an epidermal inclusion cyst on histopathology

An epidermal inclusion cyst arises from an obstructed hair follicle. On mammogram, there is a well-defined lesion, which is continuous with the skin [Figure 6].

### Tuberculosis

**Figure 7 (A,B):**
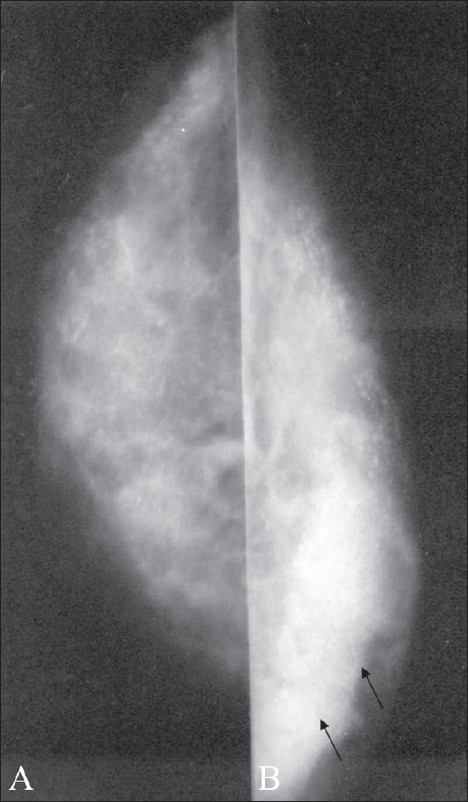
Tuberculosis. Craniocaudal views of the right (A) and the left (B) breast show a normal right breast with an ill-defined mass in the left breast (arrow). This turned out to be tuberculosis on a fineneedle aspiration biopsy

As in women, it is very difficult to differentiate breast tuberculosis from carcinoma breast, both clinically and radiologically. An ill-defined mass may be seen on mammography [Figure 7]. Diagnosis can only be made on fine needle aspiration biopsy.

### Breast cancer

Male breast cancer accounts for <1% of total male breast lesions.[5–6] The most common presentation is as a palpable mass. Because there is a paucity of parenchyma as compared with the female breast, the malignancy rapidly progresses to the next stage, with the appearance of secondary signs like nipple retraction, fixation to deeper tissues, skin ulceration or adenopathy.

**Figure 8 (A,B):**
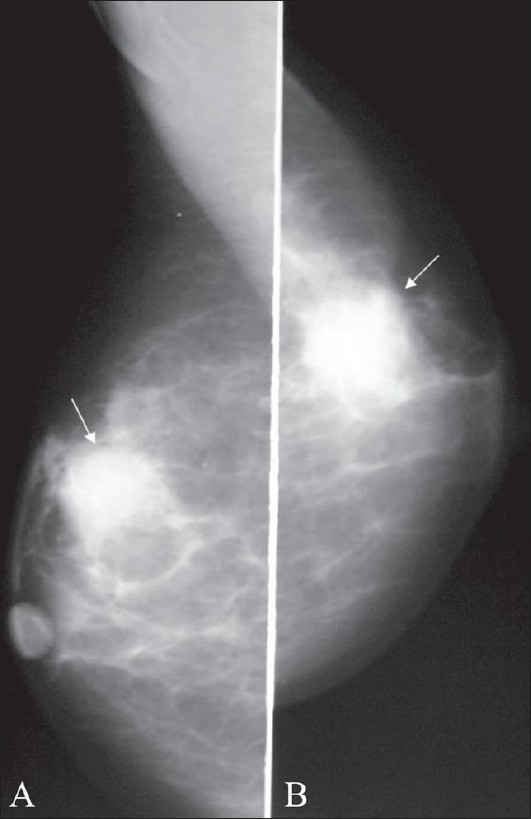
Ductal carcinoma: Mediolateral (A) and craniocaudal (B) views show a well-defined lesion with lobulated margins (arrows)

**Figure 9 (A,B):**
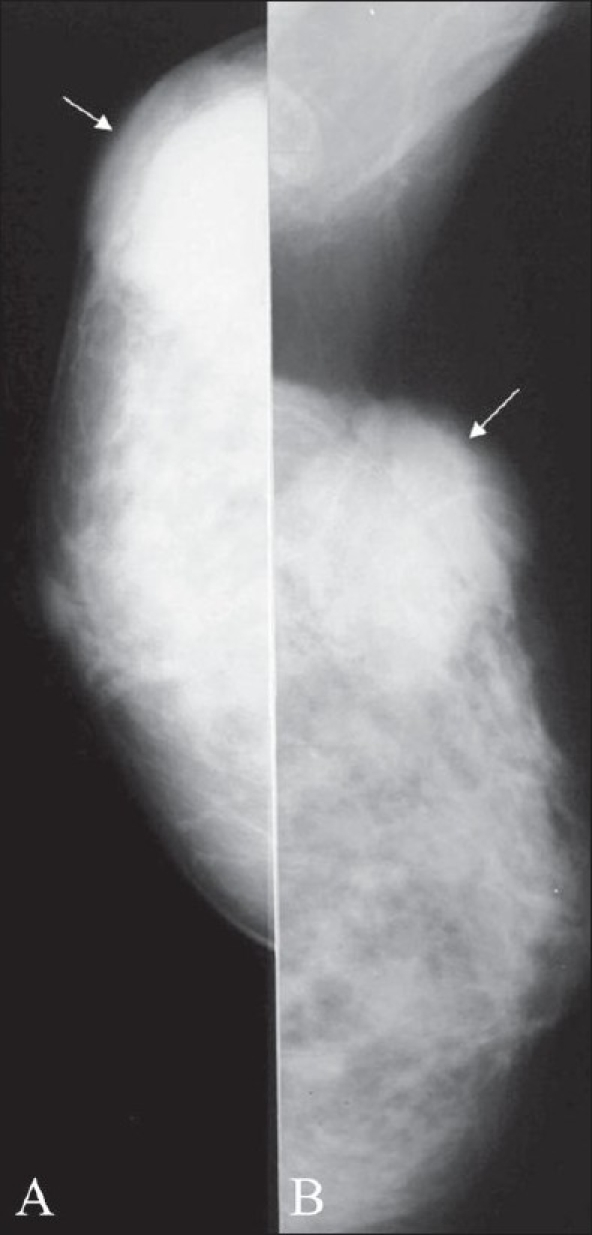
Ductal carcinoma: Craniocaudal (A) and mediolateral (B) views show an ill-defined mass with spiculated margins (arrows)

**Figure 10 (A,B):**
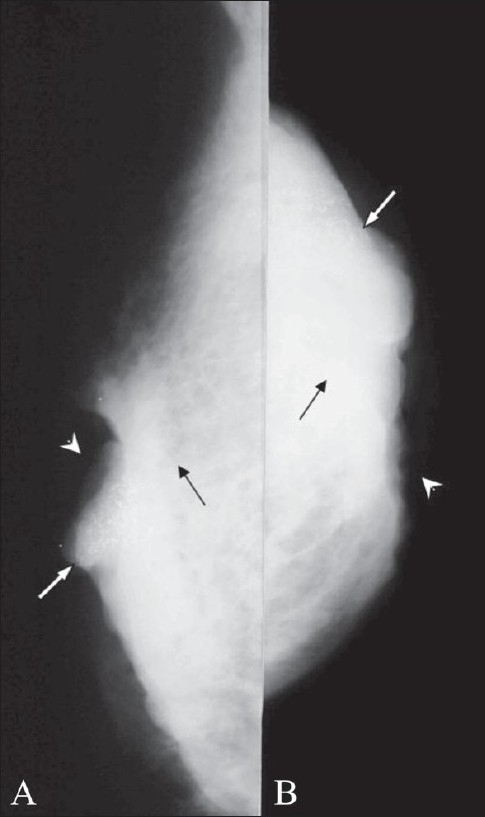
Carcinoma with secondary signs. Mediolateral (A) and craniocaudal (B) views show a mass (black arrows) with involvement of the overlying skin with ulceration (arrowheads) and calcification (white arrows)

On mammography, the mass is placed eccentrically. The margins of the lesions can be well circumscribed, lobulated or spiculated [Figures 8,9]. Secondary signs may be seen [Figure 10].

To conclude, the majority of male breast lesions are benign. Differentiation between benign and malignant masses is critical. Mammography has been shown to be an accurate method for distinguishing benign gynecomastia from carcinoma breast. Absence of a well-defined mass and secondary signs favor the diagnosis of gynecomastia, whereas an eccentrically located mass, the presence of secondary features and advanced age of the patient suggest carcinoma breast.
